# The Relationship Between Electrocardiographic Findings and Cardiac Magnetic Resonance Results in Patients with Acute Myocarditis: A Retrospective Analysis

**DOI:** 10.3390/medicina61081444

**Published:** 2025-08-11

**Authors:** Michaela Kyriakou, Nikolaos P. E. Kadoglou, Stefanos Sokratous, Elina Khattab, Christos Eftychiou, Michael M. Myrianthefs

**Affiliations:** 1 Department of Cardiology, Nicosia General Hospital, 2029 Nicosia, Cyprus; michaelakyriakou7@gmail.com (M.K.); stefanossokratous94@gmail.com (S.S.); khattab_elina@outlook.com (E.K.); chr6eft@gmail.com (C.E.); 2 Medical School, University of Cyprus, 1678 Nicosia, Cyprus; 3 Apostolos Loukas Medical Center, 2415 Nicosia, Cyprus; myr.michael@shso.org.cy

**Keywords:** acute myocarditis, ST elevation, T-wave abnormalities, CMR, LGE, myocardial edema

## Abstract

*Background and Objectives:* Electrocardiography (ECG), though non-specific, is widely applied as a valuable tool in the diagnostic work-up of acute myocarditis. Cardiac magnetic resonance (CMR) has become a key non-invasive tool. This study assessed the association of ECG findings (at baseline), echocardiographic parameters, circulating biomarkers, and CMR imaging features (myocardial edema and late gadolinium enhancement—LGE) in patients with acute myocarditis. *Materials and Methods*: This single-center, retrospective observational study included 86 patients admitted with acute myocarditis from January 2021 to December 2024. Data collected included demographics, clinical presentation, ECG, echocardiography, biomarkers (CRP, troponin I), and CMR imaging performed during hospitalization and at the six-month follow-up. Based on ECG findings, patients were stratified into three groups: no ST elevation or T-wave abnormalities (NSTG, *n* = 27), T-wave abnormalities (TWAG, *n* = 24), and ST elevation (STEG, *n* = 35). *Results*: We enrolled 86 patients (median age: 26 years; 87.2% male), and the most frequent CMR findings were either LGE (80.2%) and/or myocardial edema (75.6%). The prevalence of edema and LGE was higher in the STEG (both 91.2%) compared to TWAG (65.2%, 77.3%, respectively) and NSTG (57.7, 65.4%, respectively) (*p* < 0.05). Peak troponin levels were also higher in the STEG than other groups (*p* = 0.005). In logistic regression analysis, TWAs were independently associated with both edema (OR = 3.15, 95% CI: 1.078–9.189, *p* = 0.036) and LGE (OR = 3.93, 95% CI: 1.256–12.276, *p* = 0.019). Biomarkers were associated with lower LVEF in univariate analysis, but not in multivariate models. *Conclusions*: ECG abnormalities, particularly STE and TWA, are common in acute myocarditis and significantly associated with CMR findings. Although CMR remains essential for definitive diagnosis and risk stratification in acute myocarditis, ECG may serve as a valuable initial screening tool in the context of a multimodal diagnostic approach.

## 1. Introduction

Acute myocarditis is an inflammatory condition of the myocardium, typically caused by infectious agents (most commonly viral), autoimmune disorders, or toxins [1]. It is usually presented with a wide clinical spectrum ranging from mild, self-limiting symptoms to severe heart failure, arrhythmias, and sudden cardiac death [2]. Although its incidence is estimated at 10–22 cases per 100,000 annually [3], accurate estimation remains limited due to diagnostic challenges. Notably, it is more frequently diagnosed in young males, during the second and third decades of life [4], and accounts for 6% to 10% of sudden cardiac deaths (SCDs) in autopsy-based series [5].

Due to its heterogeneous clinical presentation, the diagnosis of myocarditis requires a multimodal approach. According to the 2013 European Society of Cardiology (ESC) position statement [6], the diagnosis of suspected myocarditis requires at least one clinical and one diagnostic criterion, in the absence of significant coronary artery disease (CAD). Endomyocardial biopsy remains the gold-standard technique for the firm diagnosis of myocarditis. However, its invasive nature and low sensitivity limit its use [7]. In contrast, cardiac magnetic resonance (CMR) imaging has emerged as a pivotal non-invasive modality, capable of analyzing the myocardial tissue texture. Techniques such as T2-weighted imaging and late gadolinium enhancement (LGE) may reveal, with high accuracy, the myocardial edema and the non-ischemic pattern of myocardial injury [8]. Hence, CMR has nowadays become an essential modality for the investigation of suspected myocarditis.

Nevertheless, the other parameters (i.e., symptoms, electrocardiography-ECG, biomarkers) clustered in the diagnostic algorithm of myocarditis play a significant role. Among them, ECG remains a first-line diagnostic tool due to its availability, low cost, and easy application. ECG changes in myocarditis are generally non-specific, common abnormalities include sinus tachycardia, ST-segment elevation (STE), and T-wave inversion (TWI) [9]. These abnormalities, while not pathognomonic, may reflect underlying myocardial inflammation, edema, or necrosis [9]. While ECG is part of the broader diagnostic process, this study does not evaluate it as an independent diagnostic tool. Instead, it aims to investigate the association between early ECG changes and established markers of myocardial injury—including CMR features and circulating biomarkers—in patients with suspected acute myocarditis based on ESC criteria. Despite its widespread use, few studies have explored how early ECG abnormalities relate to findings on CMR. Therefore, the primary aim of this retrospective study was to determine whether specific ECG features—specifically T-wave abnormalities (TWAs) and STE—are associated with myocardial edema and LGE on CMR. The secondary aims were to assess the relationship between biomarkers such as troponin I (TnI) and C-reactive protein (CRP) with the left ventricle ejection fraction (LVEF).

## 2. Materials and Methods

### 2.1. Study Design

This was a single-center, retrospective, observational study based on registry data collected from the Cardiology Department at Nicosia General Hospital from 1 January 2021 to 31 December 2024. The study was conducted in accordance with the principles of the Declaration of Helsinki, and the study protocol was approved by the national bioethics committee (NCBC 66/2024).

### 2.2. Participants

This study initially included all patients aged >14 years who were consecutively admitted to the Cardiology Department of the General Hospital of Nicosia with clinically suspected myocarditis. After thorough evaluation during hospitalization, patients were considered eligible if the diagnosis of myocarditis was established according to the criteria outlined in the European Society of Cardiology (ESC) 2013 position statement [6]. Specifically, patients were diagnosed with suspected acute myocarditis if they fulfilled at least one clinical presentation criterion (such as chest pain, arrhythmias, or unexplained heart failure) and at least one diagnostic criterion (such as elevated troponin levels, new ECG abnormalities, or cardiac imaging findings), in the absence of significant coronary artery disease (CAD), which was ruled out by either invasive or non-invasive coronary angiography.

**Figure 1:**
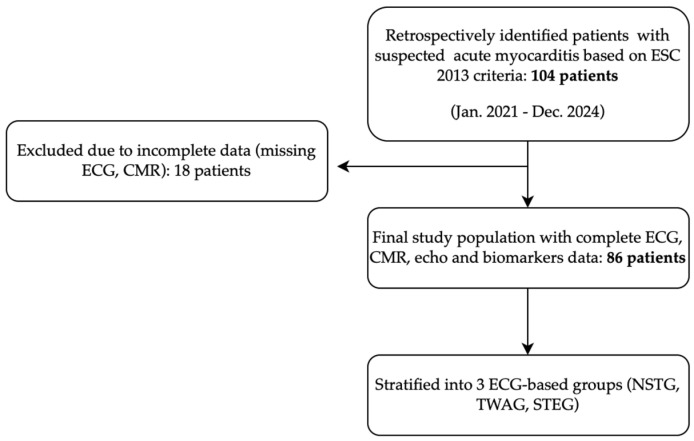
Flowchart of patient inclusion. Of 104 patients with suspected myocarditis, 86 with complete data were analyzed and stratified by ECG findings.

We retrospectively identified 104 patients diagnosed with suspected acute myocarditis over a 4-year period, based on ESC 2013 criteria [6]. Complete admission data, including both ECG and CMR, were available for 86 patients, who were included in the final analysis. The cohort was stratified into three groups based on ECG findings on admission: (1) no ST elevation or T-wave abnormalities group (NSTG), (2) T-wave abnormalities group (TWAG), and (3) ST-segment elevation (STEG) group. A flowchart is presented in Figure 1.

### 2.3. Data Collection

The following data were collected from medical records at baseline (admission and during hospitalization) and 6 months after their visits to our outpatient clinic:

- Demographic characteristics and past medical history;
- Clinical presentation and recent infection prior to hospitalization;
- Laboratory findings: TnI and CRP levels;
- ECGs;
- Imaging findings (TTE and CMR);
- Coronary angiography findings;
- Medication regimens;
- Cardiac-related events during follow-up, like acute heart failure (HF) and heart failure-related therapy up-titration.

Myocardial edema and fibrosis (LGE) were recorded as present or absent, based on formal CMR reports issued by cardiologists specialized in cardiac imaging at the cooperating centers. ECGs were analyzed by a single observer using the digital recordings.

### 2.4. Statistical Analysis

The distribution of continuous variables was assessed using the Shapiro–Wilk test. Most variables were not normally distributed, so continuous data are presented as the median and interquartile range (IQR), while categorical variables are presented as frequencies and percentages. We used the Kruskal–Wallis test for between-group comparison and the Mann–Whitney U test for post hoc analysis. Due to the absence of major clinical events and the small number of patients developing HF (*n* = 4) during the follow-up period, our analysis focused on identifying correlations of ECG with CMR markers of myocarditis (LGE and/or edema) and other parameters like the left ventricle ejection fraction (LVEF), biomarkers, etc. In univariate analysis, we used Spearman’s rank correlation coefficient to assess the association between biomarkers and LVEF. We also performed univariate logistic regression to evaluate the associations of CRP and T-wave abnormalities (TWAs) with the presence of myocardial edema and LGE on CMR. Only variables that showed statistically significant associations in univariate analysis were included in the multivariable regression models. A two-tailed *p*-value < 0.05 was considered statistically significant for all final analyses. All statistical analyses were performed using SPSS software (version 30.0; IBM Corp., Armonk, NY, USA).

## 3. Results

### 3.1. Demographic Characteristics and Past Medical History

Most patients were male (75 patients, 87.2%), and the median age was 26 years (IQR: 20–33.25 years). Baseline characteristics, including comorbidities, are summarized in Table 1. A significant proportion were active smokers (*n* = 26, 30.2%) and less of them had obesity, hypertension, or dyslipidemia. A prior history of either myocarditis, or autoimmune disease or congenital heart disease, was documented in 10 (11.6%), 5 (5.8%), and 3 patients (3.5%), respectively. January accounted for the highest percentage of admissions (13.9%), suggesting a seasonal peak in myocarditis incidence.

### 3.2. Clinical Presentation and Recent Infection Prior to Hospitalization

In addition to infection-related symptoms, almost all patients (84 patients, 97.7%) reported chest pain, while 10 patients (11.6%) reported dyspnea, and 5 patients (5.8%) reported palpitations on the top of other symptoms. No patient presented with syncope. The median length of hospital stay was 8 days (IQR: 5–10.25 days). Four patients (4.6%) presented with acute HF and cardiogenic shock in the context of fulminant myocarditis. All of them were transferred to the intensive care unit (ICU) and received intravenous inotropic support. Three of them additionally required mechanical ventilation, and one patient required mechanical circulatory support with extracorporeal membrane oxygenation (ECMO). No deaths were recorded throughout this study.

Notably, 55 patients (64%) reported infection within the month prior to presentation. Specifically, 28 patients (50.9%) reported an upper respiratory tract infection, 3 (5.5%) had a lower respiratory tract infection, and 16 (29.1%) experienced a gastrointestinal infection. Considering the potential influence of the COVID-19 pandemic on our findings, we also recorded data on recent SARS-CoV-2 infection or vaccination. Four patients (4.7%) had a confirmed SARS-CoV-2 infection, and six patients (7%) had received a COVID-19 vaccine within 30 days prior to presentation. On the other hand, 31 patients (36%) either reported other recent infections of non-specific origin or an autoimmune disease exacerbation preceded their admission.

### 3.3. Baseline Findings

#### 3.3.1. Electrocardiogram

**Figure 2:**
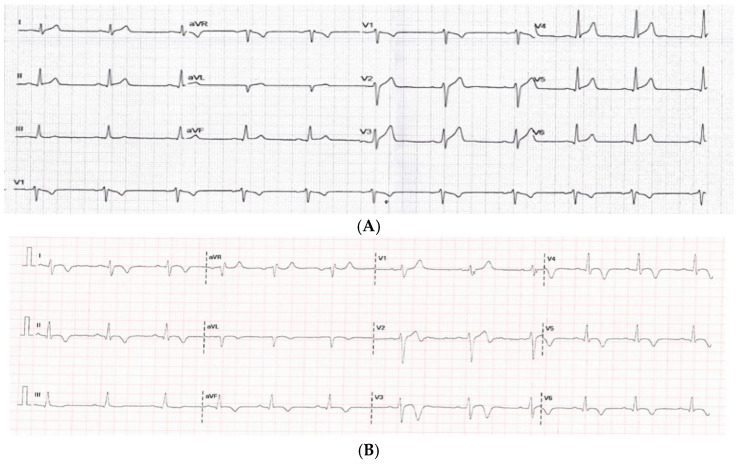
(**A**) Diffuse, concave ST elevation in leads I, aVL, II, aVF, V2–6 at admission, and ST depression in leads V1, aVR. (**B**) Inverted T waves in leads I, II, aVL, aVF, V2–6 at discharge.

The most frequently observed ECG abnormalities on admission were repolarization abnormalities, present in 59 patients (68.6%). These included T-wave abnormalities (TWAs) in 24 patients, characterized by T-wave inversion, biphasic T waves, or flattening in at least two contiguous leads, and concave ST-segment elevation (STE) in 35 patients, defined as an elevation of ≥1 mm in the ST segment in at least two contiguous leads. Over time, a clear progression of ST-segment changes was observed, with T-wave inversion becoming more prevalent by the time of discharge, particularly in the inferolateral leads. Figure 2 illustrates the evolution of electrocardiographic changes in one patient with TWI on discharge (2B). Additional ECG findings included sinus tachycardia (17.4% of patients) and paroxysmal atrial fibrillation (2.3% of patients). Notably, one patient presented with sustained ventricular tachycardia and another with complete atrioventricular block. Both individuals had a reduced LVEF and severe clinical manifestations, highlighting a potential association between severe arrhythmias and ventricular dysfunction.

#### 3.3.2. Imaging Modalities

All patients underwent TTE at the time of admission (within 24 h). The median LVEF was 60% (IQR: 52.25–60.0%). The vast majority of them (87.2%) presented with a preserved global systolic function (LVEF ≥ 50%). Regional wall motion abnormalities (RWMAs) were identified in 27.2% of patients, indicating localized myocardial systolic impairment. Global longitudinal strain (GLS) analysis was available, and most patients appeared within the normal range of values [median: −18.1% (IQR: −20.0% to −15.2%)]. Additionally, mild pericardial effusion was observed in 18 patients.

**Figure 3:**
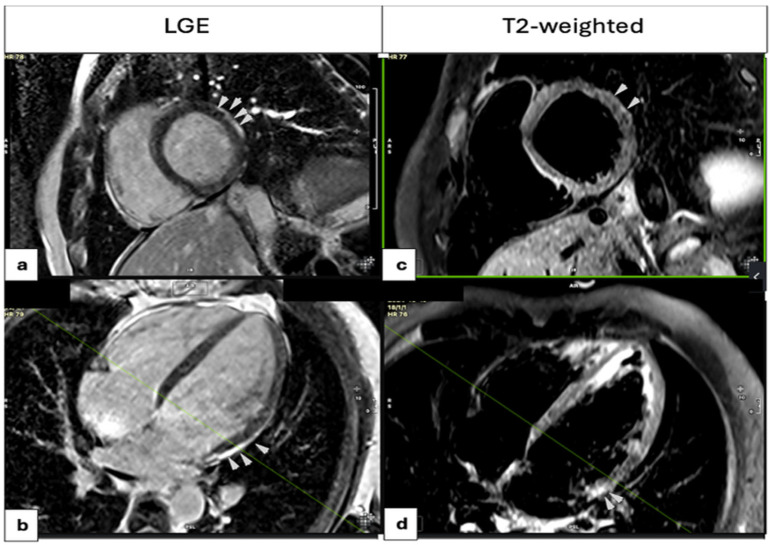
LGE distribution (**a**,**b**) and T2-weighted images (**c**,**d**). High-intensity signal (white arrows in the basal anterolateral wall segment on T2-weighted images (**c**,**d**)) indicates myocardial edema, with corresponding LGE in the same regions (**a**,**b**), findings consistent with acute myocarditis.

Following admission, CMR imaging was performed within a median period of 8 days (IQR: 5–16 days). CMR-based RWMAs were identified in less patients (13.0%) than in TTE, while a diffuse mild hypokinesia was mostly reported with a low impact on the absolute values of the LVEF, presumably due to the young age of participants. The observed discrepancy between TTE and CMR findings may also be attributed to the difference in timing between the two examinations, with TTE typically performed on the first day of hospitalization and CMR on or around the eighth day. Given the dynamic nature of acute myocarditis, evolving myocardial function over time could explain this variation. Fibrosis was defined by the presence of LGE on CMR, which was detected in 69 patients (80.2%), primarily localized to the subepicardial region of the basal and mid segments of the inferolateral and lateral walls (Supplementary Figure S1). The midwall distribution and transmural involvement of LGE were uncommon (7.0% and 5.8% of cases, respectively). Myocardial edema was assessed using T2-weighted (T2W) imaging and was present in 65 patients (75.6%). Early gadolinium enhancement and mild pericardial effusion were infrequently observed (9.9% and 22.2% of patients). Other rare findings were the right ventricular thrombus (1 patient), pleural effusion, and underlying lung abnormalities (1 patient). Figure 3 illustrates the distribution of LGE and corresponding T2-weighted imaging findings. Of the patients, 34 underwent invasive coronary angiography, while the rest received coronary computed angiography to rule out CAD.

#### 3.3.3. Biomarkers

The median CRP level (normal value in our laboratory: <5 mg/L) increased from 49.4 mg/L (IQR: 14.22–83.5 mg/L) at admission to 60.12 mg/L (IQR: 23.8–100.0 mg/L) at the peak and then decreased to 8.76 mg/L (IQR: 5.0–17.9 mg/L) at discharge. A similar pattern was observed in median TnI levels (cut-off: <0.5 ng/mL), with values of 2.53 ng/mL (IQR: 0.79–4.84 ng/mL) on admission, peaking at 8.66 ng/mL (IQR: 2.32–9.68 ng/mL), and declining to 0.09 ng/mL (IQR: 0.03–0.48 ng/mL) at discharge.

#### 3.3.4. Comparison Based on ECG Findings on Admission

To assess the association between ECG presentation on admission and other parameters, the cohort was stratified into three groups based on ECG findings: (1) no STE or TWA group (NSTG, *n* = 27), (2) TWA group (TWAG, *n* = 24), and (3) STE group (STEG, *n* = 35). A Kruskal–Wallis test revealed a statistically significant difference in peak TnI levels between groups (*p* = 0.005). A post hoc analysis showed considerably higher peak TnI values in the STEG compared to the NSTG (*p* = 0.007), implicating an association between STE and a greater burden of myocardial injury. Similarly, the prevalence of myocardial edema and late gadolinium enhancement (LGE) was significantly higher in the STEG and TWAG groups compared to the NSTG (*p* = 0.008 and *p* = 0.049, respectively), highlighting a strong association between CMR findings and ECG repolarization abnormalities at admission. Similarly, the prevalence of myocardial edema and LGE was significantly higher in the STEG and TWAG compared to the NSTG (*p* = 0.008 and *p* = 0.049, respectively), indicating a strong association between CMR markers and ECG repolarization abnormalities on admission. These results suggest a progressively greater extent of myocardial injury and inflammation in patients with more pronounced ECG changes, particularly STE. No statistically significant differences were found between groups for age, TnI on admission or discharge, CRP levels (admission, peak, or discharge), GLS, echo-based LVEF, and CMR-based LVEF and RVEF (all *p* > 0.05). The results are summarized in Table 2.

### 3.4. Follow-Up

#### 3.4.1. Clinical Outcomes and ECG and Imaging Findings

During the 6-month follow-up period, one patient experienced a relapse of myocarditis requiring re-admission. Although some patients complained about episodes of significant chest pain (*n* = 3) or palpitations (*n* = 7), no new hospitalization was deemed necessary.

At the end of follow-up, 25 patients exhibited TWI, 5 had persistent concave STE indicating benign early repolarization, and 1 had ST-segment depression.

The repeated TTE after 6 months showed a modest but statistically significant improvement in LVEF over time [median increase: 3.0% (IQR: 0.0–10.0%), *p* < 0.001]; however, this change is likely not clinically relevant. Due to technical reasons, we had a limited dataset available for repeated measurements of GLS, and therefore, we did not include it in the analysis during follow-up. All patients underwent follow-up CMR imaging at 6 months, which revealed residual LGE in 82.5% of cases. Only one patient continued to show signs of myocardial edema, indicating ongoing active inflammation.

#### 3.4.2. Therapy

Most of our patients received pharmacological therapy primarily based on standard international HF guidelines [10]. We mainly delivered a tailored therapy for a reduced LVEF or hypertension (ACE inhibitors) and symptom relief (like β-blockers for palpitations, or ACE inhibitors/diuretics for dyspnea). Those medications were prescribed during hospitalization and continued until the 6-month follow-up. If symptoms were absent and cardiac systolic function was normal, the medications were then discontinued. Antivirals, antibiotics, or corticosteroids for autoimmune disease were prescribed by physicians only when they were deemed necessary. Colchicine was administered to five patients (5.8%) for 14–28 days for coexistent pericarditis. The pharmacologic therapy during hospitalization and follow-up is presented in Supplementary Table S1.

### 3.5. Correlations

In univariate analysis, CRP levels on admission and at the peak were significantly associated with a lower LVEF (*p* = 0.005; *p* = 0.001, respectively). Regarding myocardial injury markers, admission TnI levels were inversely associated with the LVEF (*p* = 0.048), while peak TnI levels were not significantly associated with the LVEF (*p* = 0.185). However, those correlations were lost in multivariate analysis. When considering myocardial edema and LGE on CMR, univariate analysis showed that both CRP levels at admission and the presence of TWA were associated with these findings. However, in multivariate logistic regression analysis, only the TWA remained an independent determinant of myocardial edema (*p* = 0.036) or LGE (*p* = 0.019) on CMR at baseline. The results are summarized in Table 3 and Table 4.

## 4. Discussion

Our initial aim was to assess the prognostic value of the baseline findings. However, the absence of adverse events and small number of patients with HF and a reduced LVEF in our cohort prevented a meaningful evaluation of the prognosis. Given the central role of CMR imaging in the diagnosis of myocarditis, we focused on exploring correlations between CMR findings—specifically myocardial edema and LGE—and both ECG repolarization abnormalities and biochemical markers. This single-center, retrospective analysis highlights the clinical relevance of ECG findings and inflammatory biomarkers in detecting myocardial injury. Notably, our main finding is the significant association between specific ECG changes—particularly T-wave abnormalities (TWAs)—and the presence of myocardial edema and LGE on CMR, both of which are hallmark indicators of myocardial inflammation and injury.

Our findings are consistent with previous registries regarding gender distribution, type of symptoms, and the seasonal pattern observed in acute myocarditis presentation [6]. Clinically, most cases follow a mild course with a low likelihood of deterioration requiring advanced supportive therapies such as ECMO, as demonstrated in our cohort. Since that was a retrospective study, the impact of pharmaceutical therapy on the clinical course was not evaluated.

A potential association between the LVEF and systemic inflammation (CRP levels) as well as myocardial injury (TnI levels) was observed in univariate analysis. However, those associations did not remain significant in multivariate analysis, indicating that neither CRP nor TnI was independently associated with the LVEF in our cohort. Our findings align with the 2013 ESC position statement, which highlights that while elevated cardiac troponin (cTn) indicates myocardial injury, it does not reliably reflect disease severity or predict clinical outcomes in acute myocarditis [6]. Supporting this, a recent study reported that although hs-cTn was universally elevated in patients with myocarditis, it did not correlate with the degree of LV systolic dysfunction [11]. Furthermore, in our cohort, the CRP levels on admission were no longer significantly associated with myocardial edema and LGE on CMR in multivariate logistic regression where TWA was included as a covariate. This is consistent with the findings of Berg et al. [12], who reported that cardiac enzymes and inflammatory markers alone do not reliably predict the presence or extent of LGE. However, Goitein et al. [13] observed a positive correlation between CRP levels and the extent of LGE, though not with cTn. Overall, in patients with acute myocarditis, the prognostic role of biomarkers of inflammation and myocardial injury is under investigation and perhaps there are many confounders (like timing of when samples are obtained) blurring their contribution to diagnosis and prognosis work-up.

ECG is usually non-pathognomonic [9] and characterized by relatively low sensitivity (~47%) and uncertain specificity [14]. However, it remains a widely available, easily repeatable, low-cost, and non-invasive diagnostic tool. Integrated into a multimodal diagnostic approach, it could serve as an attractive tool for the initial approach of patients with suspected myocarditis. We observed that patients with clinical manifestations of myocarditis and concomitant distinct ECG abnormalities on admission—particularly TWA and STE—demonstrated greater myocardial involvement on CMR. This was reflected by a higher prevalence of myocardial edema and LGE compared to patients without these ECG changes. Importantly, TWA was independently associated with the presence of myocardial edema and LGE on multivariate analysis. This suggests that early ECG findings may reflect underlying myocardial inflammation and injury, even prior to advanced imaging, underscoring their clinical relevance. Notably, the prevalence of CMR abnormalities did not differ significantly between patients with STE and those with TWA. In contrast, patients without ECG changes showed a markedly lower prevalence of CMR-detected abnormalities and no significant association with myocardial edema or LGE. Therefore, ECG changes on admission may be sensitive to myocardial insult. From a practical perspective, these findings support the potential utility of ECG for early risk stratification. Patients presenting with TWA or STE may benefit from earlier cardiac MRI and more intensive clinical monitoring, allowing for the timely diagnosis and management of myocardial injury.

STE is the most common ST abnormality in myocarditis [9], reported in 24% to 73% of cases [15,16,17]. Variability reflects differences in presentation timing relative to symptom onset [9]. In most cases, STE resolves rapidly—within 24 to 48 h [15,16,17]—a trend also observed in our cohort. Myocarditis-induced STE is typically diffuse, involving multiple limb and precordial leads, while sparing aVR and V1, which may show reciprocal depression [18,19]. Based on underlying pathophysiological mechanisms, several investigators have proposed that the presence of STE on admission may be associated with a more severe or adverse disease course. Notably, Nucifora et al. demonstrated that specific ECG patterns—particularly the magnitude and persistence of ST-segment elevation, along with the development of TWI—were significantly correlated with the extent of myocardial damage on CMR [15]. Furthermore, a study by Ramantauskaitė et al. (2024) [20] found that patients with STE patterns on ECG had significantly higher CRP levels and lower LVEFs compared to those without STE, suggesting a more severe inflammatory response and impaired cardiac function. These observations underscore the potential of STE as an indicator of more extensive myocardial involvement and a more adverse clinical course in myocarditis.

TWI is a common electrocardiographic finding in acute myocarditis [21], with variable prevalence (9% to 48%)—depending on the population studied and the timing of ECG acquisition [15,16,22]. In our cohort, 24 patients demonstrated TWI at admission, with the number rising significantly during hospitalization to a total of 59 cases. Indeed, we observed symmetrical TWI, particularly in leads that previously exhibited STE, which may reflect evolving myocardial injury or recovery. The underlying mechanisms of TWI may represent myocardial inflammation-induced repolarization heterogeneity [9,23]. Specifically, inflammation can prolong the action potential duration in the epicardial layer of the myocardium [9]. This alteration leads to a reversal in the normal sequence of ventricular repolarization, resulting in TWI on the ECG. In a healthy heart, repolarization typically begins in the epicardial layer and concludes in the endocardial layer, producing a positive T wave. However, in cases of transmural or epicardial myocarditis, the prolonged action potential in the epicardial layer causes an “anti-dromic” repolarization direction, starting from the endocardial layer and ending at the epicardial layer, formulating a TWI [9]. This phenomenon reflects the heterogeneity in repolarization induced by myocardial inflammation, which is a hallmark of myocarditis [23]. This mechanistic link is further supported by the findings of De Lazzari et al. [24], who demonstrated that transmural myocardial edema is the strongest independent predictor of TWI. Their study revealed a significant association between TWI and both myocardial edema (median 5 vs. 3 segments; *p* = 0.015) and LGE (median 4 vs. 3 segments; *p* = 0.002), with a concordance rate of 88% between the ECG localization of TWI and areas of myocardial edema on CMR. Notably, despite its association with myocardial inflammation, TWI was not correlated with a reduced LVEF at the baseline and at the six-month follow-up. Furthermore, Zareba et al. found that a low T-wave amplitude or negative T-waves are independently associated with myocardial fibrosis, as evidenced by LGE or an elevated extracellular volume fraction (ECV) on CMR. In their study, TWA was more prevalent in patients with myocardial fibrosis compared to those without (66% vs. 42%, *p* < 0.001), and multivariable analysis confirmed this association after adjusting for clinical covariates [25]. These findings underscore the clinical significance of TWA in myocarditis, highlighting the role of ECG not only in guiding the need for urgent imaging but also in early risk stratification of myocarditis severity.

In addition to the repolarization abnormalities analyzed in our study, other ECG features such as QRS fragmentation have been proposed as potential markers of myocardial fibrosis in myocarditis. Ferrero et al. [26] reported that fragmented QRS may correlate with the presence and topographic distribution of LGE on CMR, reflecting underlying myocardial scarring. Moreover, they identified fragmented QRS as a possible prognostic marker, as its persistence at follow-up was associated with contractile dysfunction. Although QRS fragmentation was not assessed in our cohort, its potential diagnostic and prognostic significance warrants consideration in future studies.

CMR plays a central role in the evaluation of myocarditis, especially when endomyocardial biopsy is not feasible. It enables comprehensive tissue characterization, detecting edema, hyperemia, necrosis, and fibrosis. The Lake Louise Criteria (LLC), updated in 2018, recommend using at least one T2-based marker (for edema) and one T1-based marker (e.g., LGE or elevated ECV) to confirm myocarditis and enhance diagnostic precision [27]. However, its sensitivity is highly time-dependent: inflammation may evolve or resolve over days to weeks [28,29], necessitating optimal timing—ideally within 7 to 14 days of symptom onset—to capture active changes [30]. In our study, CMR was performed at a median of 8 days post-admission, successfully identifying myocarditis in over 80% of cases through a predominantly subepicardial distribution of LGE, and/or myocardial edema on T2-weighted imaging, confirming active inflammation.

Despite the resolution of symptoms and inflammatory markers at follow-up, persistent LGE was observed in 82.5% of patients, indicating chronic structural myocardial damage. This residual fibrosis provides an arrhythmogenic substrate and may predispose one to future ventricular dysfunction [31,32]. Notably, the presence, extent, and location of LGE carry prognostic significance. A meta-analysis by Georgiopoulos et al. demonstrated that the presence of LGE was associated with a significantly higher risk of adverse outcomes (pooled hazard ratio 3.28; 95% CI: 1.69–6.39; *p* < 0.001), and anteroseptal LGE localization was independently linked to a worse prognosis (hazard ratio 2.58; 95% CI: 1.87–3.55) [33].

## 5. Limitations and Future Directions

This study has several important limitations. First, its retrospective, single-center design inherently limits the generalizability of our findings to broader populations. As with all retrospective analyses, missing or incomplete data may introduce bias and limit the strength of statistical inferences. Incomplete electrocardiographic (ECG) data in some cases further restricted the ability to capture the full temporal evolution of ECG changes, potentially underestimating the incidence of transient or late-onset abnormalities. Another limitation is the imbalance between comparison groups, in the size of the comparison groups, particularly among the three ECG-defined subgroups, which may introduce bias and reduce the statistical power of comparisons. Smaller groups may be more susceptible to the effects of outliers or random variation, which can impact the reliability and generalizability of the findings. In addition, we could not draw conclusions about the direct prognostic value of ECG findings since most of our participants received ACE inhibitors and b-blockers. Finally, the evolving definitions of myocardial infarction—including the concepts of type 2 MI and MINOCA (myocardial infarction with non-obstructive coronary arteries)—pose increasing diagnostic challenges in distinguishing acute myocarditis from ischemic syndromes. While our retrospective study design limited our ability to fully account for these entities, we acknowledge that this diagnostic overlap is clinically relevant and should be addressed in future prospective studies.

To build upon these findings, future research should prioritize prospective, multicenter studies to enhance the validity of our results and minimize selection bias. Standardized protocols for data collection, ECG interpretation, and imaging analysis would reduce variability and increase the sensitivity and specificity of the diagnostic methods. The routine incorporation of advanced myocardial strain imaging techniques, such as speckle-tracking echocardiography, could further improve early the detection of subclinical myocardial dysfunction and enhance risk stratification. Also, extending the duration of follow-up beyond six months is also crucial to better characterize the natural history of residual myocardial fibrosis, monitor the risk of late arrhythmic events, and assess the progression to dilated cardiomyopathy. A larger, prospective study with a longer follow-up period would also be better powered to clarify the prognostic value of ECG and other imaging parameters.

## 6. Conclusions

ECG abnormalities—particularly ST-segment elevation and T-wave inversion—are common in acute myocarditis and show meaningful correlations with myocardial inflammation and fibrosis on CMR. Regarding the latter as the diagnostic gold standard, ECG may serve as a valuable and widely accessible tool for initial screening and monitoring of myocardial injury and inflammation. By highlighting these associations, our findings aim to enhance the diagnostic value of ECG and support the development of more integrated, multimodal diagnostic strategies for patients with acute myocarditis.

## Figures and Tables

**Table 1:** Baseline characteristics.

Sex	Male, *n*	75 (87.2%)
	Female, *n*	11 (12.8%)
Age		26 (20–33.25)
Comorbidities— ASCVD risk factors	Smoking, *n*	26 (30.2%)
	Obesity, *n*	3 (3.5%)
	Hypertension, *n*	3 (3.5%)
	Dyslipidemia, *n*	6 (7%)
	Family history of ASCVD, *n*	7 (8.1%)
	Diabetes, *n*	0 (0%)
Other comorbidities	Prior myocarditis, *n*	10 (11.6%)
	Autoimmune disease, *n*	5 (5.8%)
	Congenital heart disease, *n*	3 (3.5%)

ASCVD, atherosclerotic cardiovascular disease; *n*, number of patients.

**Table 2:** Comparison based on ECG findings at admission.

Variable	NSTG (*n* = 27)	TWA (*n* = 24)	STEG (*n* = 35)	*p*-Value
Age (years)	26 (19–34)	28.5 (21.3–34)	25 (20–32)	0.77
TnI admission (ng/mL)	1.8 (0.6–3.7)	1.8 (0.9–4.6)	3.0 (1.1–9.0)	0.066
TnI peak (ng/mL)	3.7 (1.4–9.0)	4.3 (1.5–7.6)	7.1 (4.6–19.3)	0.005
TnI discharge (ng/mL)	0.15 (0.04–0.80)	0.08 (0.03–0.34)	0.09 (0.03–0.29)	0.709
CRP admission (mg/L)	27.3 (6.4–88.6)	47.8 (6.8–80.5)	62.2 (26.4–92.3)	0.215
CRP peak (mg/L)	27.3 (7.0–86.6)	52.8 (27.9–108.4)	65.7 (36.2–102)	0.18
CRP discharge (mg/L)	6.6 (2.3–15.0)	10.8 (5.0–18.8)	10.2 (6.0–18.5)	0.332
LVEF—Echo (%)	60 (58–60)	57.5 (51.3–60)	57 (50–60)	0.09
GLS (%)	−18.3 (−19.3 to −17.4)	−18.2 (−19.6 to −13.0)	−18.0 (−20.7 to −15.0)	0.915
LVEF—CMR (%)	58 (55–60)	58 (53–60)	60 (57–60)	0.537
RVEF—CMR (%)	54.5 (52–59.8)	53.5 (49.8–57.8)	54.5 (50–56.5)	0.785
T2W (Edema) (*n*)	15 (57.7%)	15 (65.2%)	31 (91.2%)	0.008
LGE (*n*)	17 (65.4%)	17 (77.3%)	31 (91.2%)	0.049

Values are presented as median (interquartile range); *n*, number of patients (%, percentage of patients). CMR, cardiac magnetic resonance; CRP, C-reactive protein; GLS, global longitudinal strain; LGE, late gadolinium enhancement; LVEF, left ventricular ejection fraction; NSTG, group with no ST-segment or T-wave abnormalities; RVEF, right ventricular ejection fraction; STEG, ST-segment elevation group; TnI, Troponin I; TWAG, T-wave abnormalities group; T2W, T2-weighted images.

**Table 3:** Logistic regression analysis of predictors of myocardial edema on CMR.

Variable	Univariable		Multivariable	
	OR (95% CI)	*p*	OR (95% CI)	*p*
CRP	1.4 (1.05–2.05)	0.041	1.11 (1.02–1.76)	0.473
TWA	4.11 (1.21–10.77)	<0.001	3.15 (1.078–9.19)	0.036

CRP, C-reactive protein; TWA, T-wave abnormality; OR = odds ratio.

**Table 4:** Logistic regression analysis of predictors of LGE on CMR.

Variable	Univariable		Multivariable	
	OR (95% CI)	*p*	OR (95% CI)	*p*
CRP	1.26 (1.02–1.98)	0.032	1.22 (1.05–2.25)	0.446
TWA	5.55 (1.99–15.78)	<0.001	3.93 (1.256–12.276)	0.019

CRP, C-reactive protein; TWA, T-wave abnormality; OR = odds ratio.

## Data Availability

Data is unavailable due to privacy or ethical restrictions.

## Supplementary Materials

The following supporting information can be downloaded at: https://www.mdpi.com/article/10.3390/medicina61081444/s1, Figure S1: Localization of subepicardial LGE according to AHA classification. Table S1: Pharmacologic therapy during hospitalization and follow-up.

## Abbreviations

The following abbreviations are used in this manuscript:

ACE	Angiotensin-Converting Enzyme
AHA	American Heart Association
ARB	Angiotensin II Receptor Blocker
ASCVD	Atherosclerotic Cardiovascular Disease
CI	Confidence Interval
CMR	Cardiac Magnetic Resonance
CRP	C-Reactive Protein
cTnI	Cardiac Troponin I
Echo	Echocardiography
ECG	Electrocardiogram
ECMO	Extracorporeal Membrane Oxygenation
ECV	Extracellular Volume
ESC	European Society of Cardiology
hs-cTn	High-Sensitivity Cardiac Troponin
GLS	Global Longitudinal Strain
ICU	Intensive Care Unit
IQR	Interquartile Range
LGE	Late Gadolinium Enhancement
LLC	Lake Louise Criteria
LVEF	Left Ventricular Ejection Fraction
MRA	Mineralocorticoid Receptor Antagonist
NSTG	Group with No ST Elevation or T-Wave Abnormalities
OR	Odds Ratio
RVEF	Right Ventricular Ejection Fraction
RWMA	Regional Wall Motion Abnormality
SCD	Sudden Cardiac Death
STE	ST Elevation
STEG	ST Elevation Group
T2W	T2-Weighted Images
TTE	Transthoracic Echocardiography
TWAG	T-Wave Abnormalities Group
