# Supplementary material for: The Relationship Between Electrocardiographic Findings and Cardiac Magnetic Resonance Results in Patients with Acute Myocarditis: A Retrospective Analysis
Source: Medicina (Kaunas). 2025 Aug 11;61(8):1444. doi: 10.3390/medicina61081444 (PMC12388294; doi:10.3390/medicina61081444)
Supplement: Supplementary file 1 [file medicina-61-01444-s001.zip › medicina-3714358-supplementary.pdf]

Supplementary Materials

Supplementary Figure S1. Localization of subepicardial LGE according to AHA classification.

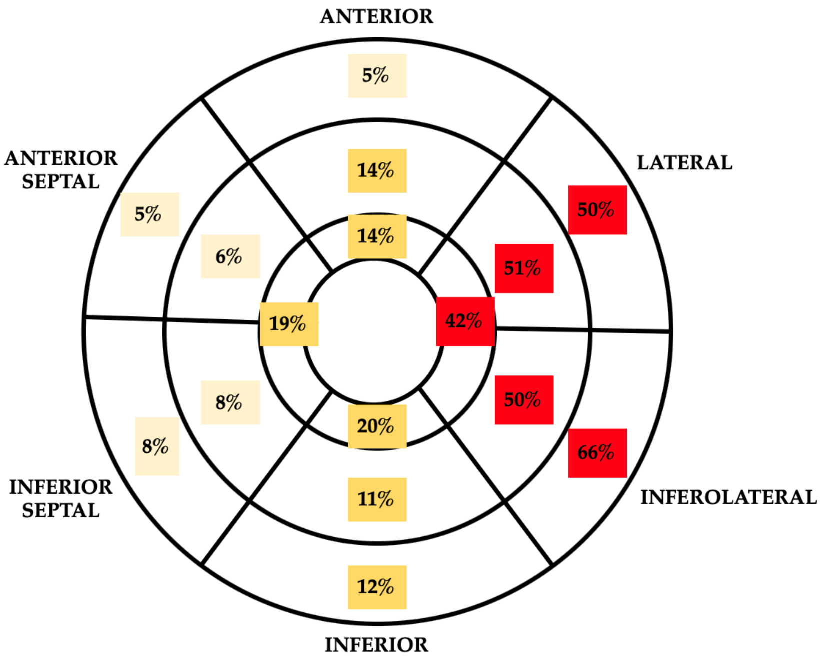

Table S1. Pharmacologic therapy during hospitalization and follow-up

Beta-blockers	68 (79.0%)
ACE inhibitors	58 (67.4%)
Both beta-blockers and ACE inhibitors	54 (62.8%)
ARB	1 (1.2%)
MRA	2 (2.3%)
Loop diuretics	2 (2.3%)
Colchicine	5 (5.8%)
Antibiotics	6 (7%)
Corticosteroids	4 (4.6%)

Values expressed in numbers, n and percentages, %. ACE, Angiotensin-converting enzyme; ARB, Angiotensin receptor blocker; MRA, Mineralocorticoid receptor antagonist.
